# Correlation between nutritional, hematological and infectious characteristics and classification of the type of epidermolysis bullosa of patients assisted at the Dermatology Clinic of the Hospital Universitário de Brasília*

**DOI:** 10.1590/abd1806-4841.20154575

**Published:** 2015

**Authors:** Márcia Carolline dos Santos Sousa, Carmen Dea Ribeiro de Paula, Pedro Luiz Tauil, Izelda Maria Carvalho Costa

**Affiliations:** 1 Universidade de Brasília (UnB) - Brasília (DF), Brazil

**Keywords:** Child nutrition, Epidermolysis bullosa, Infection

## Abstract

Epidermolysis bullosa comprises a group of phenotypically different genodermatosis, hereditary or acquired, characterized by skin fragility and subsequent formation of blisters in response to mechanical trauma, and which may also affect mucous membranes. This study aimed to analyze the relation between the nutritional, hematologic, infectious characteristics and the type of epidermolysis bullosa, through a descriptive case study based on data from medical records of 10 patients with epidermolysis bullosa assisted regularly at the Dermatology Clinic of the Hospital Universitário de Brasília. The old classification of the type of epidermolysis bullosa, weight and height, blood count, white blood cell count, platelet count and description of the type and frequency of secondary infections during the service were considered. We verified a predominance of iron deficiency anemia, chronic leukocytosis, thrombocytosis, chronic malnutrition, low height for age and thinness, and people with epidermolysis bullosa simplex exhibited appropriate relation between height/age and BMI/age. The non-specific skin infection was the most prevalent in both sexes. The severity of the type of epidermolysis bullosa and frequency of secondary infections did not form a directly proportional relation. The absence of direct proportion in all cases between the type of epidermolysis bullosa and the analysis parameters suggest a possible significant interference from other aspects such as the extent of the affected skin area, extracutaneous type of engagement and specific genetic mutation. The inclusion of these factors in the new classification proposed by Fine et al can contribute significantly to a better correlation of clinical parameters and appropriate preventive and therapeutic approaches.

Epidermolysis bullosa (EB) is a group of phenotypically different genodermatosis, hereditary or acquired, characterized by skin fragility and subsequent formation of blisters in response to mechanical trauma, which may also affect mucous membranes.^1-3^ Due to the severe forms of disease and its high risk of infection, the mortality rate between affected newborns is high.^1-3^ Children with EB may suffer delayed growth and puberty, which often are linked to low food intake, justified by the difficulty in swallowing or anemia.^4^ EB is a rare disease with great variability in incidence and prevalence, and, in Brazil, epidemiological data are poorly understood.^3^

According to the recent proposal of Fine et al, the classification of EB can be compared with the layers of an onion, and the first step is the inclusion within one of the four major groups according to the place of formation of blisters: intraepidermal (EB simplex), inside (junctional EB), below the basement membrane zone (dystrophic EB), and with a mixed pattern (Kindler syndrome).^5^ The next step considers the phenotypic characteristics such as distribution (localized or generalized) and severity of cutaneous and extracutaneous involvement.^5^ The third step is based on the transmission mode and is identifiable by the specific gene involved, determined by means of immunohistochemical techniques and mutation analysis.^5^

This work aims to contribute to the analysis of the relation between the nutritional, hematologic, and infectious characteristics, as well as the type of EB, which can provide better therapeutic approach and quality of life for patients and their caregivers.

This is a descriptive case study based on data from medical records of 10 patients with EB, assisted regularly at the Dermatology Clinic of the Hospital Universitário de Brasilia. The old classification of the type of EB, weight and height, blood count, white blood cell count, platelet count and description of the type and frequency of secondary infections throughout the service were considered. Tables 1, 2 and 3 correlate patients and their respective data.

**Table 1:** Age (years) during the survey period (March 2013), sex, type of EB, total time of assistance (years) and total infections in patients with epidermolysis bullosa followed at the Dermatology Clinic of the Hospital Universitário de Brasília (HUB)

Patient	Age	Sex	Type of EB	Time	Total infections
P1	19	F	Recessive dystrophic	19	0
P2	8	F	Recessive dystrophic	9	3
P3	7	M	Simplex	7	7
P4	11	M	Recessive dystrophic	12	2
P5	12	F	Recessive dystrophic	3	2
P6	8	M	Simplex	8	2
P7	18	M	Recessive dystrophic	19	10
P8	14	F	Recessive dystrophic	15	21
P9	13	F	Junctional or dystrophic	12	16
P10	15	F	Junctional or dystrophic	10	1

Male (M); Female (F).

**Table 2:** Weight (kg), height (m), BMI (kg/m2) at the end of the survey period (March 2013) and relations between height and age (H/A) and body and body mass index and age (BMI/A) in patients with epidermolysis bullosa followed at the Dermatology Clinic of the Hospital Universitário de Brasília (HUB)

Patient	Age	Sex	Weight	Heigh	BMI	H/A	BMI/A
P1	19	F	27	1,5	12	< p3	< p3
P2	8	F	16,3	1,15	12,32	< p3	< p3
P3	7	M	29,7	1,38	15,59	p97	< p15-50
P4	11	M	22,9	1,36	12,38	p3-15	< p3
P5	12	F	20,9	1,31	12,17	< p3	< p3
P6	8	M	30,2	1,43	14,76	p97	< p15-50
P7	18	M	35,2	1,61	13,57	< p3	< p3
P8	14	F	17,2	1,22	11,55	< p3	< p3
P9	13	F	25,7	1,28	15,68	< p3	< p3
P10	15	F	41,8	1,6	16,32	p15-50	< p3

BMI: body mass index

**Table 3:** Distribution of infections recorded since the start of the care of patients until the end of the survey period (March 2013) of patients with epidermolysis bullosa followed at the Dermatology Clinic of the Hospital Universitário de Brasília (HUB)

Patient	URI	PA	Moniliasis	Otitis	PNM	Skin infection	Myiasis	UTI	Balanoposthitis	AGEC	Oxyuriasis
P1	0	0	0	0	0	0	0	0	0	0	0
P2	0	0	0	0	0	3	0	0	0	0	0
P3	0	0	0	0	0	7	0	0	0	0	0
P4	0	0	1	0	0	1	0	0	0	0	0
P5	1	0	0	0	0	5	0	0	0	0	0
P6	0	0	0	0	0	2	0	0	0	0	0
P7	0	0	0	1	1	6	1	0	1	0	0
P8	0	3	2	1	2	8	0	3	0	2	0
P9	0	0	0	0	0	11	1	4	0	0	0
P10	0	0	0	0	0	0	0	0	0	0	1

URI: upper airway infection; PA: Pharyngoamigdalitis; PNM: Pneumonia ; UTI: Urinary tract infection; AGEC: acute gastroenterocolitis

There was a predominance of chronic malnutrition, low height for age and thinness, and patients with EB simplex presented appropriate relation between height/age and BMI/age. Also, there was a predominance of cases of iron deficiency anemia, chronic leukocytosis and thrombocytosis without direct correlation to the type of EB. Nonspecific skin infection was the most prevalent in both sexes. Severity of the type of EB and frequency of secondary infections did not form a directly proportional relation.

Absence of direct proportion in all cases between the type of EB and analysis parameters suggest a possible significant interference from other aspects such as the extent of the affected skin area, extracutaneous involvement and type of specific genetic mutation. Inclusion of these factors in the new classification proposed by Fine et al can contribute significantly to a better correlation of clinical parameters and appropriate preventive and therapeutic approaches.

Due to the rarity of the disease, the number of cases studied was small and further studies are needed to better evaluate the data found.
